# Can the Thermal Runaway of a Thionyl Chloride Cells Generate a Shock Wave?

**DOI:** 10.1002/gch2.202500140

**Published:** 2025-06-26

**Authors:** Juliette Charbonnel, Sébastien Dubourg, Etienne Testard, Thibaut Rochard, Christophe Magnier, David Brun‐Buisson, Rémi Vincent

**Affiliations:** ^1^ University Grenoble Alpes CEA LITEN DEHT Grenoble F‐38000 France; ^2^ University Grenoble Alpes University Savoie Mont Blanc CNRS Grenoble INP LEPMI Grenoble F‐38000 France; ^3^ CEA DAM Le Ripault Monts F‐37260 France

**Keywords:** cell, shock wave, thionyl chloride

## Abstract

Due to their high energy density, thionyl chloride technology are used in a wide range of applications in the aerospace industry. These cells consist of a lithium metal anode and a *SOCl*_2_ liquid cathode. The safety problems posed by these cells received particular attention in the 70 and 80s. However, the generation of shock waves during thermal runaway has never been demonstrated. In this article, for the second time, aerial shock waves are characterized for cells composed of lithium metal. Although the TNT equivalent of thionyl chloride cells is widely dispersed between 0.008 and 0.3 g. Its impact on the mechanical structures of the battery module should not be neglected, nor should its impact on people.

## Graphical abstract

This study presents results obtained from abusive tests conducted on primary thionyl chloride batteries. The safety of these batteries, which are used in numerous aerospace industry applications, received particular attention in the 1970 and 1980s. However, the risks of explosions with shock wave formation has never been demonstrated until now.

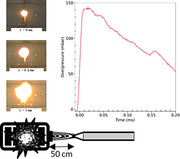

## 1 Introduction

The thionyl chloride cells are used in space applications due to their high energy density, their ability to function in extreme environments up to − 40 °C and their reliability.^[^ ^1^, ^2^, ^3^ ^]^

It is a liquid cathode battery. The anode is made of a lithium‐metal foil with a current collector made of nickel or stainless steel. The liquid cathode is made of *SOCl*_2_ with a current collector with a porous carbon composition. To prevent short‐circuits, a passivation layer made of *LiCl* is formed on the lithium‐metal surface. During the discharged, the lithium is gradually oxidized. Then, the lithium ions react with the chlorine ions and formed *LiCl* which is deposited in the porosity of the carbon electrode. This reaction also formed sulphur ions and *SO*_2_ which are soluble in *SOCl*_2_ and formed *SOCl*_2_.^[^ ^4^, ^5^, ^6^ ^]^

Unfortunately, these primary batteries could lead to thermal runaway (TR). TR could be defined as “a process of uncontrolled heat released and rapid temperature rise”.^[^ ^7^ ^]^ From the late 70s, Dey et al. emphasized that the TR of thionyl chloride battery could lead to an explosion under short‐circuit, heating, and overdischarge abuses.^[^ ^6^, ^8^, ^9^, ^10^ ^]^ Furthermore, it seems that using a safety device such as a vent did not significantly cool the cell and consequently stop TR.^[^ ^10^ ^]^ Differential thermal analysis (DTA) were carried out on thionyl chloride battery materials. Lithium‐metal DTA shows an exothermic peak at 180 °C and two exothermic peaks at 195 °C and 202 °C. According to the DTA made by A. Dey an explosion is highlighted whenever *SOCl*_2_ is one of the compounds. The addition of lithium metal has been demonstrated to induce a blast explosion, as indicated by the DTA.

It seemed that the thionyl chloride battery could have an explosion behavior during TR.^[^ ^5^ ^]^ However, the safety studies for this technology do not determine whether the explosion is a detonation or a deflagration by measuring the overpressure released during TR. A deflagration is usually confined and slower than the speed of sound contrary to a detonation which is usually no confined and faster than the speed of sound. It is important to determine whether the thermal runaway of a cell can lead to the formation of a detonation, as the shock wave produced by the detonation induces a pressure front locally that is particularly damaging to mechanical structures. In this study, open‐air overpressure measurements were carried out to better understand the explosion behavior of thionyl chloride battery during TR. Specific pressure sensors have been used to observed blast wave, especially the pressure discontinuity at shock front. The TR of thionyl chloride battery will be also recorded by fast imaging. The setup described in our previous paper has been reused.^[^ ^11^ ^]^ The experimental setup has been developed for the measurement of overpressure in the open air. The cell is then placed on the support with its axis parallel to the ground and at the same height as the Piezotronics pen‐type pressure transducers, which are positioned at different distances from the samples. The high‐speed camera was positioned perpendicularly relative to the positive terminal to observe the gases and ejecta. The Kinney and Graham law of equivalence has been used to determine the mass equivalent of trinitrotoluene (TNT) from the suppression peak.^[^ ^12^ ^]^

Four thionyl chloride cells from Saft and RS have been studied in this article. The TR has been triggered by overcharging at 2C or by overheating. The TR has been recorded by a fast camera to determine the reaction kinetic. It has also been recorded by pen‐style pressure sensor to identify the formation of a shock wave during TR by measuring aerial overpressure.

## 2 Results

### 2.1 TR Observation by Fast Imaging

**Figure 1:**
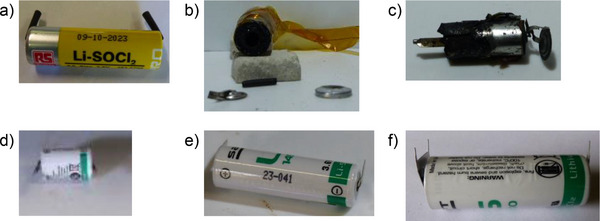
Images of (a) RS cell before TR, b) RS1 cell after TR, c) RS2 cell after TR, d) LS1 cell before TR, e) LS2 cell before TR, and (f) LS3 cell before TR.

The TR of the primary thionyl chloride RS1 cell has been triggered by overheating. The RS1 cell before TR could be seen in **Figure** **1**a. After TR, the positive pole and the casing are uncoupled (Figure 1b).

The TR of primary batteries: RS2, LS1, LS2, and LS3 cells have been triggered by overcharging. The RS2 cell before TR could be seen in Figure 1a. After TR, a third of RS2 cell has been destroyed (Figure 1c). The LS2 and LS3 cells have been almost destroyed during TR and the LS1 cell has been partially destroyed.

**Figure 2:**
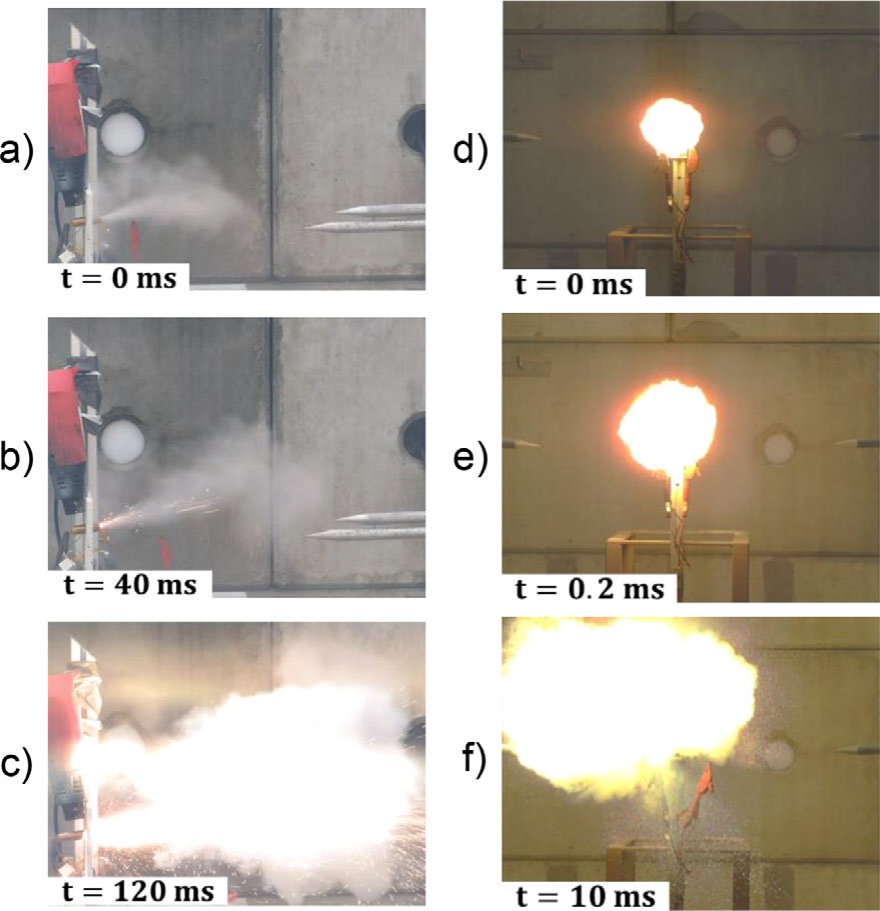
Images of (a–c) RS1 cell during overheat abuse and (d–f) RS2 cell during TR during overcharge abuse.

For the RS1 cell, its thermal runaway is trigger by an external overheating. The TR begins with smoke released from the cell, probably due to the catholyte vaporization (**Figure** **2**a). 40 ms later, sparks are expelled by the cell (Figure 2b). Finally, a fireball expands 120 ms after TR begins (Figure 2c). The TR lasts 150 ms. It was observed that cells which had been subjected to overheating did not generate any aerial overpressure waves. This is also the case for the primary thionyl chloride cells from Saft.

**Figure 3:**
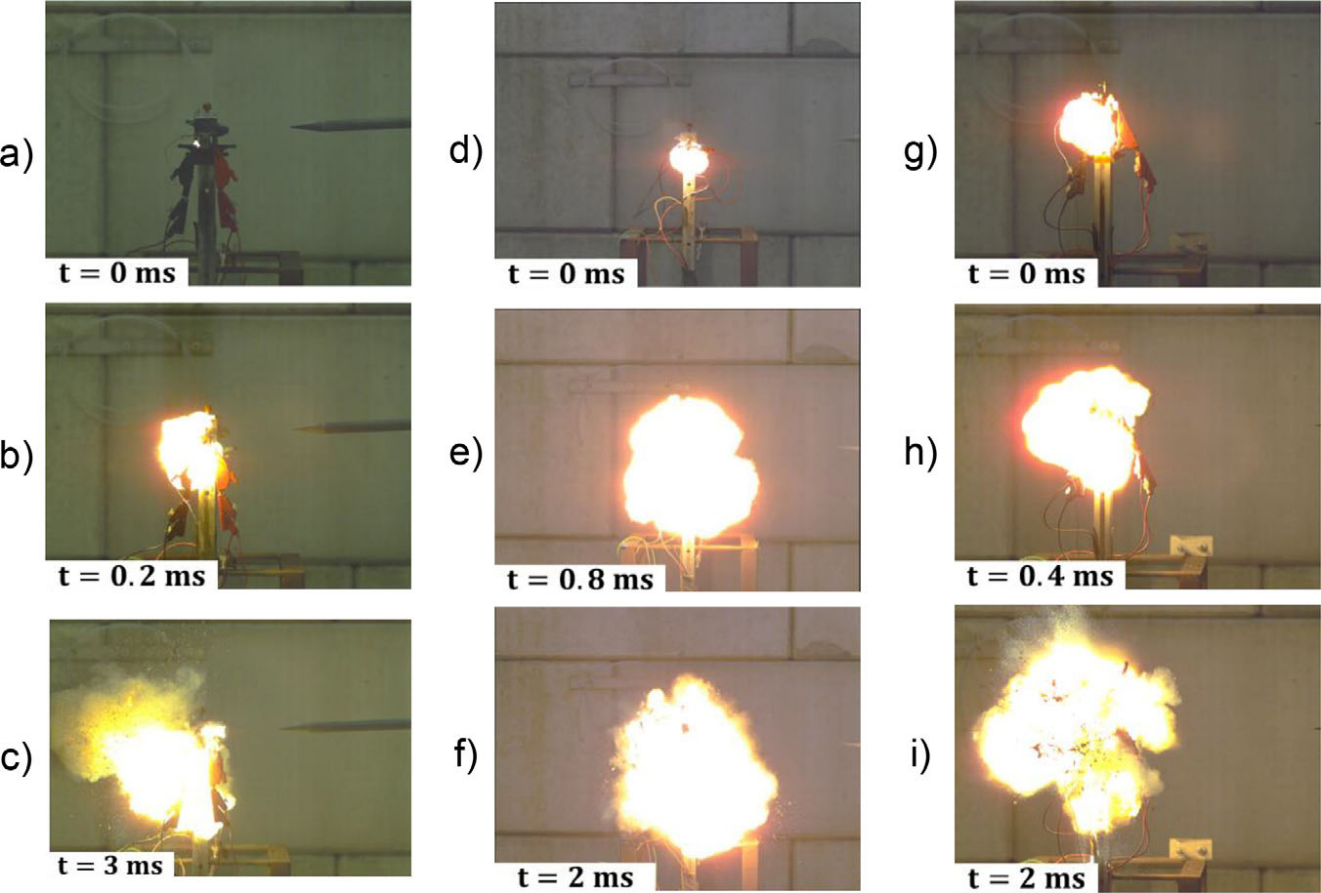
Images during overcharge abuses for (a–c) LS1 cell, d–f) LS2 cell, and (g–h) LS3 cell.

For RS2, LS1, LS2 and LS3 cells the TR is trigger by a 2C overcharge and it starts with the formation of a fireball (Figure 2d and **Figure** **3**a,d,g). Then, this fireball expands for less than 1 ms (Figure 2e and Figure 3b,e,h). For RS2 and LS1 cells, the fireball burns out respectively after 10 and 3 ms (Figures 2f and Figure 3i). For the LS2 and LS3 cells, their casing and jelly roll are broken in several pieces 2 ms after the start of TR (Figure 3f,i). The TR lasts less than 10 ms for the cells: RS2, LS1, LS2 and LS3. The TR of RS1, LS1, LS2, and LS3 cells could be watched respectively in S1, S2, S3, and S4 (Supporting Information). These primary batteries have an extremely fast reaction kinetic less than 10 ms under overcharge abuse. This extremely rapid kinetic has led to the idea of a shock wave forming during their TR.

### 2.2 Overpressure Measurement

The observation of thermal runaway with a high‐speed camera has shown that thermal runaway kinetics are extremely fast, less than 10 ms for RS2, LS1, LS2, and LS3 cells when the TR is trigger by overcharge. However, for the primary thionyl chloride cell, when the TR is triggered by an external overheating, its kinetics is slower, ≈150 ms. Overpressure measurements were carried out during the TR of RS1, RS2, LS1, LS2, and LS3 to demonstrate shock wave formation during TR.

**Figure 4:**
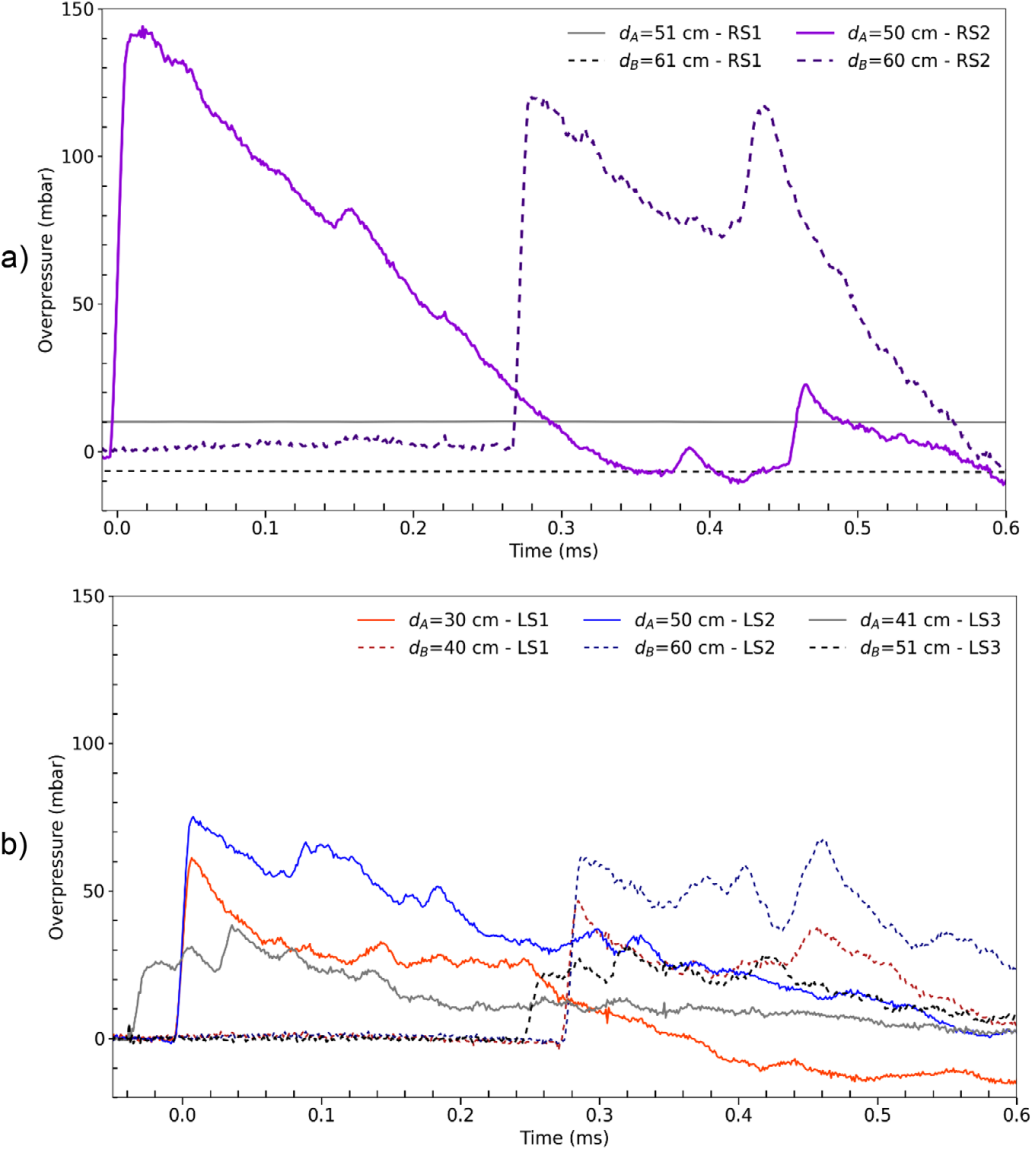
Overpressure measurements for (a) RS1 and RS2 cells and (b) LS1, LS2 and LS3 cells. *d_A_* and *d_B_* correspond to those shown in Figure 7.

**Figure** **4** shows a clear pressure discontinuity that appeared with the shock front for both RS2, LS1, LS2, and LS3 cells. This behavior is similar to that of the explosives tested in our previous article.^[^ ^11^ ^]^ In addition with fast imaging, this sharp pressure variation demonstrates that an extremely rapid reaction kinetics. The maximal overpressures are 144, 60, 69, and 42 mbar respectively for RS2, LS1, LS2 and LS3 cells (Figure 4 and **Table** **1**). Furthermore, the maximal speeds are 382, 355, 353 and 335 m.s^−1^ for RS2, LS1, LS2 and LS3 cells (Table 1). For the RS2, LS1, and LS2 cells, the overpressure waves generated are slightly supersonics. However, overpressure in cell RS1 gives a flat signal (Figure 4a). When TR is trigger by an external overheating, there is no shock wave. It is the same for the same for the primary thionyl chloride cells from Saft. On the contrary, if TR is triggered by a 2C overcharge, there is a shock wave.

**Table 1:** $p_{max}^{0}$, *m_TNT_* and *v* for LS1, LS2, LS3 and RS2 cells.

Model	$p_{max}^{0}$ (mbar)	*m_TNT_* (mg)	*v* (m.s^−1^)
RS2	144	300	382
LS1	60	8	355
LS2	74	64	357
LS3	40	11	337

As RS2, LS1, LS2, and LS3 cells show a shock front, an equivalent TNT could be determined using a law of equivalence. Kinney and Graham established a scaling law and tabulated pressure impulse, peak overpressure, and other wave parameters for explosive reaction in air.^[^ ^13^ ^]^ This law is based on the reduced distance *Z*(m.kg^−1/3^). This parameter is determined from the distance from the center of the explosion *d*(m) and the mass of equivalent TNT *m_TNT_* (kg) (Equation 1).

(1) $$Z=\frac{d}{m_{TNT}^{1/3}}$$

**Figure 5:**
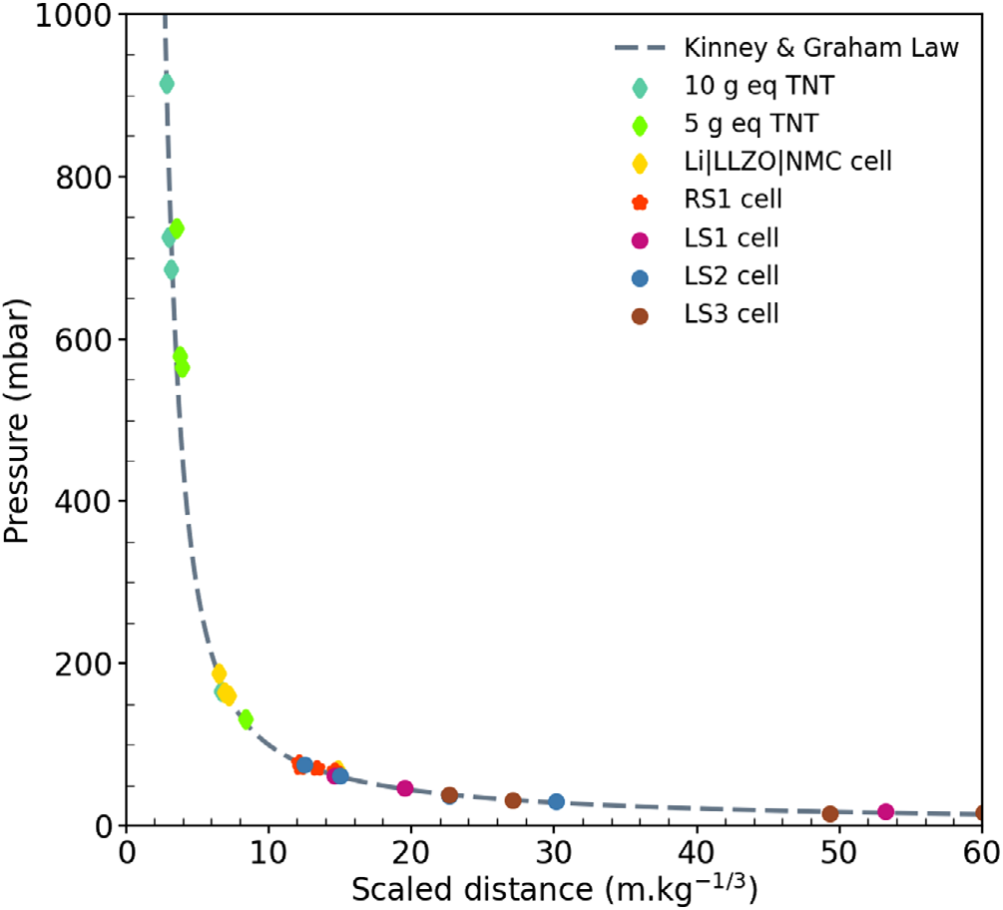
Comparison of aerial overpressure measurements as a function of scaled distance for LS1, LS2, LS3, and RS2 cells.

The scaling law of the overpressure in function of the reduced distance validated with three trials with explosive in our previous paper.^[^ ^11^ ^]^ This law of equivalence will be used to determine the equivalent TNT for the RS2, LS1, LS2, and LS3 cells (**Figure** **5** A random mass was given to each cell. Using Equation 1), a value of *Z* and the corresponding tabulated overpressure. Then, the error between the tabulated and measured overpressured was minimized for the four sensors. The equivalent masses of TNT are 300, 8, 66, and 21 mg respectively for RS2, LS1, LS2, and LS3 cells (Figure 5 and Table 1).

## 3 Discussion

Thionyl chloride cells have been abused by overheating and overcharge. For both type of abuse, the TR lasts less than 150 ms. These kinetics are fast, 3 to 4 times faster than for LG−HG2 cells.^[^ ^14^ ^]^ For the first time, it has been highlighted the formation of shock waves for thionyl chloride cells under 2C overcharge abuse.

**Figure 6:**
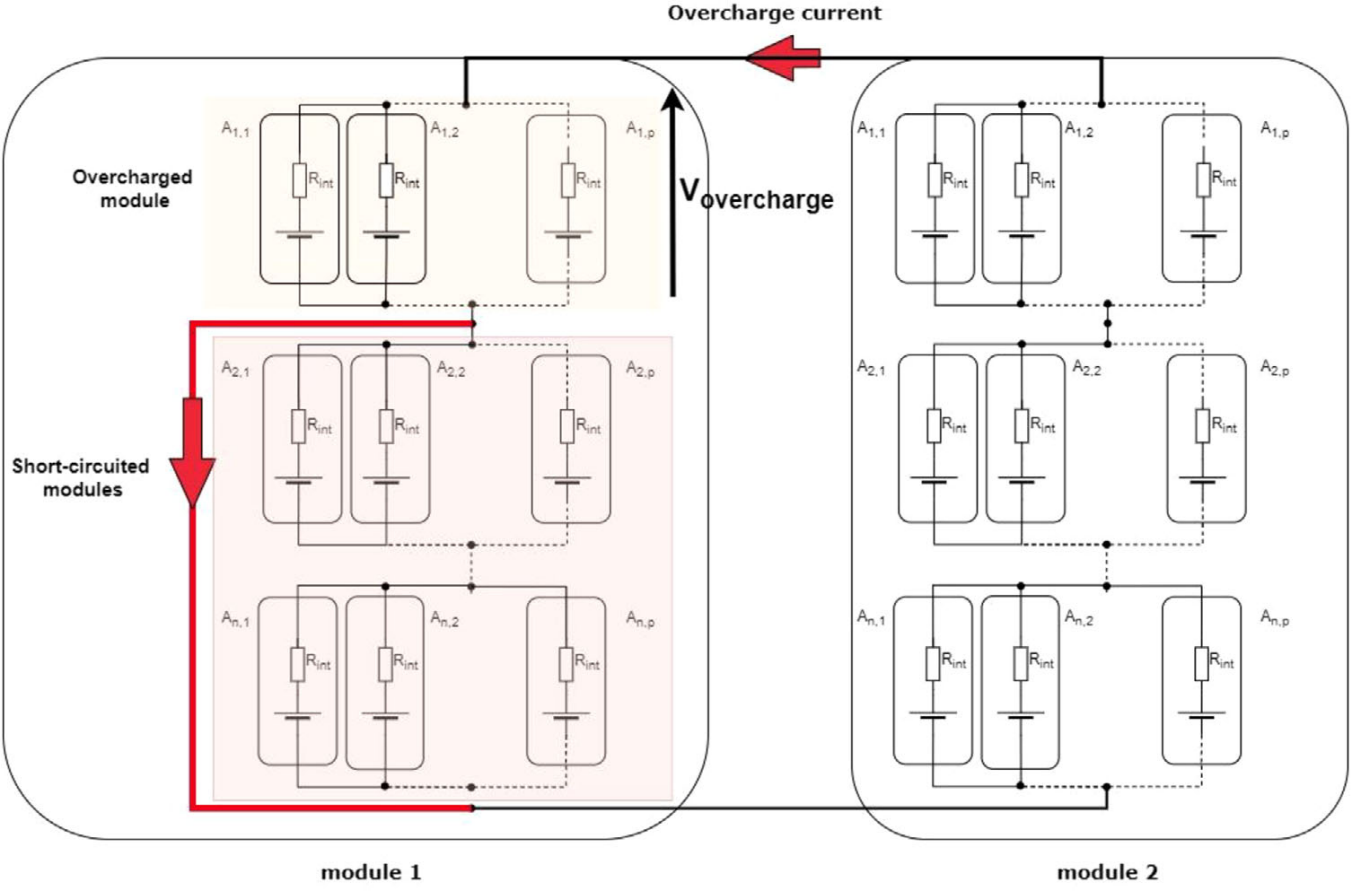
Failure that could lead to overcharging of the primary batteries of a module level.

Thionyl chloride cells are primary batteries, which, of course, are not intended to be charged. However, types of battery module or pack design, rapid overcharging is possible in the event of a failure. For example, when two battery modules are placed in parallel, if following a failure one level is short‐circuited, then the cells composing the other levels of this same module will be overcharged by the module positioned in parallel, as shown in **Figure** **6**.

A thermal runaway leading to detonation and thus to the formation of a shock wave is not a trivial matter. If the peak overpressure reaches 140 mbar, houses may suffer moderate damage and people may be injured by shards of glass and debris.^[^ ^15^ ^]^ If the peak overpressure reaches 690 mbar, even reinforced concrete buildings were severely damaged and almost everyone was killed.^[^ ^15^ ^]^

The thionyl chloride cells have a much lower TNT equivalent per cell mass than the lithium‐metal all‐solid‐state cells tested in our previous paper.^[^ ^11^ ^]^ The TNT equivalent measured for thionyl chloride technology is a maximum of 0.3 g in the case of the RS2 cell, whereas for all‐solid‐state cells, a TNT equivalent of 2.7 g had been measured. However, this explosive behaviour must be taken into account, for example, to design the casing or the pack. As for all‐solid‐state batteries, the thionyl chloride cells do not have a liquid electrolyte. This means both reaction products: cathodic and anodic active materials are very close together which leads to a direct reaction.

At 180 °C the lithium‐metal melts. The fusion of lithium‐metal leads to a greater reactivity between the lithium‐metal and the catholyte *SOCl*_2_. This high reactivity probably leads to the formation of a shock wave with the formation of *Li*_2_ *O*, *Li*_2_ *S* and *LiCl*.^[^ ^16^ ^]^

As shown in Figure 6, cases of overcharge are possible and following this overcharge the risk of thermal runaway producing a detonation is not negligible. From now on, this point should be considered during the design of the modules, in order to identify the best positioning of the fuses to limit the occurrence of this risk as much as possible.

## 4 Conclusion

For the second time, a thermal runaway leading to detonation and thus to the formation of an aerial shock wave was measured on cells composed of lithium metal. In our previous publication, it involved all‐solid‐state cells recomposed from lithium metal/LLZO/NMC, which resulted in a TNT equivalent of 2.7 g. In the case of the results presented in this publication, a maximum TNT equivalent of 0.3 g was obtained for the RS2 cell (thionyl chloride).

It is interesting to note that only these two types of cells led to the formation of shock waves following abusive tests. The very rapid reaction kinetics, necessary for the formation of a shock wave, thus seem to be favored by direct reactions that are not disrupted by the presence of additional components, which could cause secondary parasite reactions. For example, in the case of the thionyl chloride cell, the direct reaction occurs between lithium metal and thionyl chloride, or for the all‐solid‐state cell, the reaction happens between the oxygen released by the cathode and lithium metal. In the future, it will be relevant to study cells that can exhibit the same type of behavior, such as cells composed of sodium or potassium metal.

More specifically, for thionyl chloride technology, it should also be noted that there is no real correlation between the capacity of the cells and the value of the TNT equivalent. The RS cell had a much higher value than the SAFT cells for a capacity of 2.4 Ah, the latter being in the middle of the three SAFT cells tested. This indicates that the structure of the cell or part of its composition, such as the passivation layer for example, could have a significant influence on the formation of shock waves during thermal runaway. It would therefore be interesting, in a future study, to investigate many cell types with different formats, capacities, and compositions. In addition, a study with cells at different discharge levels would also be relevant to identify a possible reduction in risk over the life of the cell.

### 4.1 Methodology

#### 4.1.1 Cell

Four commercial primary thionyl chloride cells were tested. All cells have a nominal voltage of 3.6 V. The Saft LS14250 cell (LS1 cell), LS14500 cell (LS2 cell), and LS17500 cell (LS3 cell) have respectively a nominal capacity of 1.2 Ah, 2.6 Ah, and 3.6 Ah (**Table** **2**). The RS ER14505 cells: RS1 and have a nominal capacity of 2.4 Ah (Table 2).

**Table 2:** Primary cells specifications.

Model	Nominal capacity (Ah)	Nominal Voltage (V)	Abuse
RS ER14505 (RS1)	2.4	3.6	Overheating
RS ER14505 (RS2)	2.4	3.6	Overcharge
Saft LS14250 (LS1)	1.2	3.6	Overcharge
Saft LS14500 (LS2)	2.6	3.6	Overcharge
Saft LS17500 (LS3)	3.6	3.6	Overcharge

#### 4.1.2 Setup

**Figure 7:**
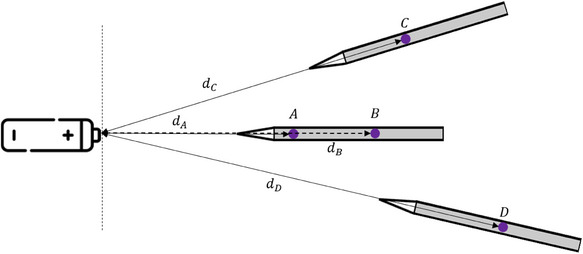
Set up of overpressure measurements with three pen‐style pressure sensors.

The primary battery studied was placed on its support parallel to the ground. A pen‐style pressure sensor was placed at the same high as the cell on its axis. This sensor is equipped with two sensing elements (**Figure** **7**A,B) placed 10 cm apart. Two other pressure sensors were place at a 30° angle on either side of the cell (Figure 7C,D). The four sensing elements are placed at various distance from the sample, between 50 and 150 cm.

The TR of each primary cell has been recorded by a high‐speed camera (Photron FAST‐CAM SA4 model 500K‐C2). The camera has been placed in front to the positive terminal to observe the ejecta and gases. A speed of 5000 frames per second (fps) was chosen as a trade‐off between image resolution and speed.

#### 4.1.3 TR Triggering Procedure

The TR has been triggered by overcharging for the LS1, LS2, LS3, and RS2 cells (Table 2) or by overtemperature for the RS1 cell (Table 2). The overcharge is obtained by applying a constant current equivalent to 2 times the cell capacity (2C) to the cell terminal until thermal runaway occurs. For the overtemperature, a heating wire is wound around the cell. To achieve thermal runaway, a constant current of 1.5 A is applied to the heating wire.

## Conflict of Interest

The authors declare no conflict of interest.

## Supporting information



Supplemental Movie 1

Supplemental Movie 2

Supplemental Movie 3

Supplemental Movie 4

## Data Availability

The data that support the findings of this study are available on request from the corresponding author. The data are not publicly available due to privacy or ethical restrictions.
